# Identification of microRNAs and their Endonucleolytic Cleavaged target mRNAs in colorectal cancer

**DOI:** 10.1186/s12885-020-06717-4

**Published:** 2020-03-23

**Authors:** Fangbin Zhou, Donge Tang, Yong Xu, Huiyan He, Yan Wu, Liewen Lin, Jun Dong, Wenyong Tan, Yong Dai

**Affiliations:** 1 Clinical Medical Research Center, The Second Clinical Medical College of Jinan University (Shenzhen People’s Hospital), 1017 North Rd Dongmen, Luohu District, Shenzhen, China; 2 Integrated Chinese and Western Medicine Postdoctoral research station, Jinan University, Guangzhou, China; 3 Department of Pathophysiology, Key Laboratory of the State Administration of Traditional Chinese Medicine, Medical College of Jinan University, Guangzhou, China; 4 Department of Oncology, Shenzhen Hospital of Southern Medical University, Shenzhen, China

**Keywords:** microRNA, Colorectal Cancer, High-throughput small RNA sequencing, Degradome sequencing, Endonucleolytic cleavage

## Abstract

**Background:**

Colorectal cancer (CRC) ranks the third among the most common malignancies globally. It is well known that microRNAs (miRNAs) play vital roles in destabilizing mRNAs and repressing their translations in this disease. However, the mechanism of miRNA-induced mRNA cleavage remains to be investigated.

**Method:**

In this study, high-throughput small RNA (sRNA) sequencing was utilized to identify and profile miRNAs from six pairs of colorectal cancer tissues (CTs) and adjacent tissues (CNs). Degradome sequencing (DS) was employed to detect the cleaved target genes. The Database for Annotation, Visualization and Integrated Discovery (DAVID) software was used for GO (Gene Ontology) and KEGG (Kyoto Encyclopedia of Genes and Genomes) pathway analysis.

**Results:**

In total, 1278 known miRNAs (clustered into 337 families) and 131 novel miRNAs were characterized in the CT and CN libraries, respectively. Of those, 420 known and eight novel miRNAs were defined as differentially expressed miRNAs (DEmiRNAs) by comparing the expression levels observed in the CT and CN libraries. Furthermore, through DS, 9685 and 202 potential target transcripts were characterized as target genes for 268 known and 33 novel miRNAs, respectively. It was further predicted that a total of 264 targeted genes for the 85 DEmiRNAs are involved in proteoglycans in cancer and the AMP-activated protein kinase signaling pathway. After systemic analysis of prognosis-related miRNA targets in those cancer-related signal pathways, we found that two targets ezrin (EZR) and hematopoietic cell-specific Lyn substrate 1 (HCLS1) had the potential prognostic characteristics with CRC regarding over survival (OS) or recurrence.

**Conclusion:**

In total, we found that endonucleolytic miRNA-directed mRNA cleavage occurs in CRC. A number of potential genes targeted by CRC-related miRNAs were identified and some may have the potential as prognosis markers of CRC. The present findings may lead to an improved better appreciation of the novel interaction mode between miRNAs and target genes in CRC.

## Background

MicroRNAs (miRNAs) represent a group of small non-coding RNAs with approximately 22 nucleotide-long, repressing the expression of complementary mRNA targets. Since first found by Lee et al. [1], miRNAs have been intensively investigated. Numerous researches have demonstrated the regulatory roles of miRNAs in tumorigenesis and development.

miRNAs inhibit the post-transcription of target mRNAs by destabilizing or degrading them. However, the mechanism of miRNAs and their target mRNA interactions between plants and animals are generally different. In plants, the endonucleolytic cleavage guided by miRNAs, rather than translational repression, decided target mRNAs’ fate [2]. Most miRNAs base pair to cleaved target mRNAs with high complementarity, which was further degraded by RNA-induced silencing complex (RISC). Numerous studies have demonstrated this plant-specific phenomenon [3–5]. However, in animals (including human), destabilization or translational repression of target mRNAs are established miRNA-targeting pathways via miRNA binding to partially complementary sites [6, 7]. Cleavage of miRNA targets is relatively rare, and only a few miRNAs exhibit this “superpower” in animals [8–12]. *HOXB8* mRNA was the first reported miRNA-directed cleavage target in mouse embryos. miR-196 represses *Hoxb8* expression by direct cleavage of complementary region with *Hoxb8* [11]. miR-249 bound to the complementary sites of the ZK637.6 transcript to guide the cleavage events in nematodes [12]. As well, mRNA cleavage guided by miRNA was found to mediate the regulation of retrotransposons and viral infection in animal [13–15]. The availability of gene-sequencing technologies made the comprehensive and systematic analysis of miRNA-induced mRNA cleavage at a whole transcriptome scale possible and degradome sequencing (DS) was then developed. Although originally designed for plants, DS was later successfully applied to animals [16].

In 2012, colorectal cancer (CRC) ranks the third among the most common malignancies globally. It accounted for approximately 10% of the estimated 14.1 million new cases of cancer [17]. Moreover, it is estimated as the second most deadly type of cancer in both females and males, with 693,600 deaths occurring annually worldwide. The roles of miRNAs in the regulation of CRC (carcinogenesis, development and tumor metastasis), through destabilization or translational repression of target mRNAs, have been widely investigated. However, whether they function by directing cleaving target mRNAs remains to be clarified [18–20]. Thus, herein, we aimed at characterizing the profile of miRNAs and their endonucleolytic cleaved target mRNAs in CRC. Colorectal cancer tissues (CTs) and adjacent tissues (CNs) from six patients with CRC were obtained to construct small RNA (sRNA) libraries for sequencing. DS was further used for the whole transcriptome analysis of miRNA-induced mRNA cleavage in CRC.

## Methods

### Study patients

Samples were surgically resected from CTs (CT3, CT4, CT5, CT6, CT7, CT8) and CNs (CN3, CN4, CN5, CN6, CN7, CN8) of six CRC patients (C3, C4, C5, C6, C7 and C8) without enteritis in Shenzhen People’s Hospital (Shenzhen, China), between August and October 2018. The six pairs of samples were immediately frozen and stored in liquid nitrogen. All patients were aged 44–62 years with histologically proven and measurable disease. Of those, C4, C6, and C7 are early-stage I-II patients (CT-E), and C3, C5, and C8 are advanced-stage III-IV patients (CT-A). Additional, details are listed in Supplementary Table 1.

### Preparation of sRNA libraries and sequencing

Total RNA was extracted from CTs and CNs with the TRIzol reagent (Invitrogen, USA). RNA was qualified and quantified by a NanoDrop 2000 (ThermoFisher, USA). cDNA constructs from the amplification of total RNA were used to prepare sRNA libraries and then sequenced on an Illumina Hiseq2500 at LC-BIO (LC Sciences, China) based on the manufacturer’s protocol.

### Characterization of known and novel miRNAs

The in-house software program, ACGT101-miR (LC Sciences, China) was applied to analysis the raw sequencing reads and redundant data such as adaptor dimers were removed according to the software’s handbook. Subsequently, known and novel miRNAs were identified by mapping the remaining clean small reads to the miRbase database (Release 22) [21]. The classification criteria for known and novel miRNAs were listed in Supplementary Table 4. In brief, sequences that perfectly matched were defined as known miRNAs, whereas sequences that were mapped to the human reference genome with no mismatches, but not matched pre-miRNAs of selected species in the miRbase were considered as novel miRNAs.

## miRNA validation

Quantitative RT-PCR (qRT-PCR) was conducted on a qTOWER 2.2 Real-Time PCR System (ANALYTIKJENA, Germany) using the aforementioned RNA samples. First-strand cDNA was synthesized using the TUREscript 1st Strand cDNA SYNTHESIS Kit (Aidlab, China) in 10-μl reaction volumes containing 0.2 mg total RNA, 2 μl 5× RT reaction mix, 0.5 μl primers, 0.5 μl TUREscript H- RTase/RI Mix and RNase-free dH2O. The reaction procedure was as follow: 25 °C for 10 min; 42 °C for 50 min and 65 °C for 15 min. A SYBR PrimeScript miRNA RT-PCR Kit (TianGen Biotech, China) was used to carry out qRT-PCR experiments. Each PCR reaction consisted of 1 μl cDNA constructs (∼100 ng), 1 μl sequence-specific forward primer, 1 μl universal reverse primer, 10 μl 2× SYBR premix EX Taq II, and 7 ml ddH2O. The samples were incubated at 95 °C for 5 min followed by 45 cycles at 95 °C for 15 s and subsequently 60 °C for 30 s. The U6 gene was used as an internal control. Each sample performed three independent biological replicates. The primers of miRNAs are listed in Supplementary Table 2.

### Degradome analysis and target identification

Total RNA samples from CTs and CNs were sent to LC-BIO for the preparation of the DS libraries. The experimental steps were as described previously [5]. The Illumina’s Cluster Station was unitized for cluster generation of the purified cDNA library and the Illumina Hiseq2500 for sequencing. The Illumina’s Pipeline and the CleaveLand 3.0 pipeline software were used to analysis the extracted reads and identify the potentially cleaved targets [22].

### Functional analysis of target genes

**Fig. 1:**
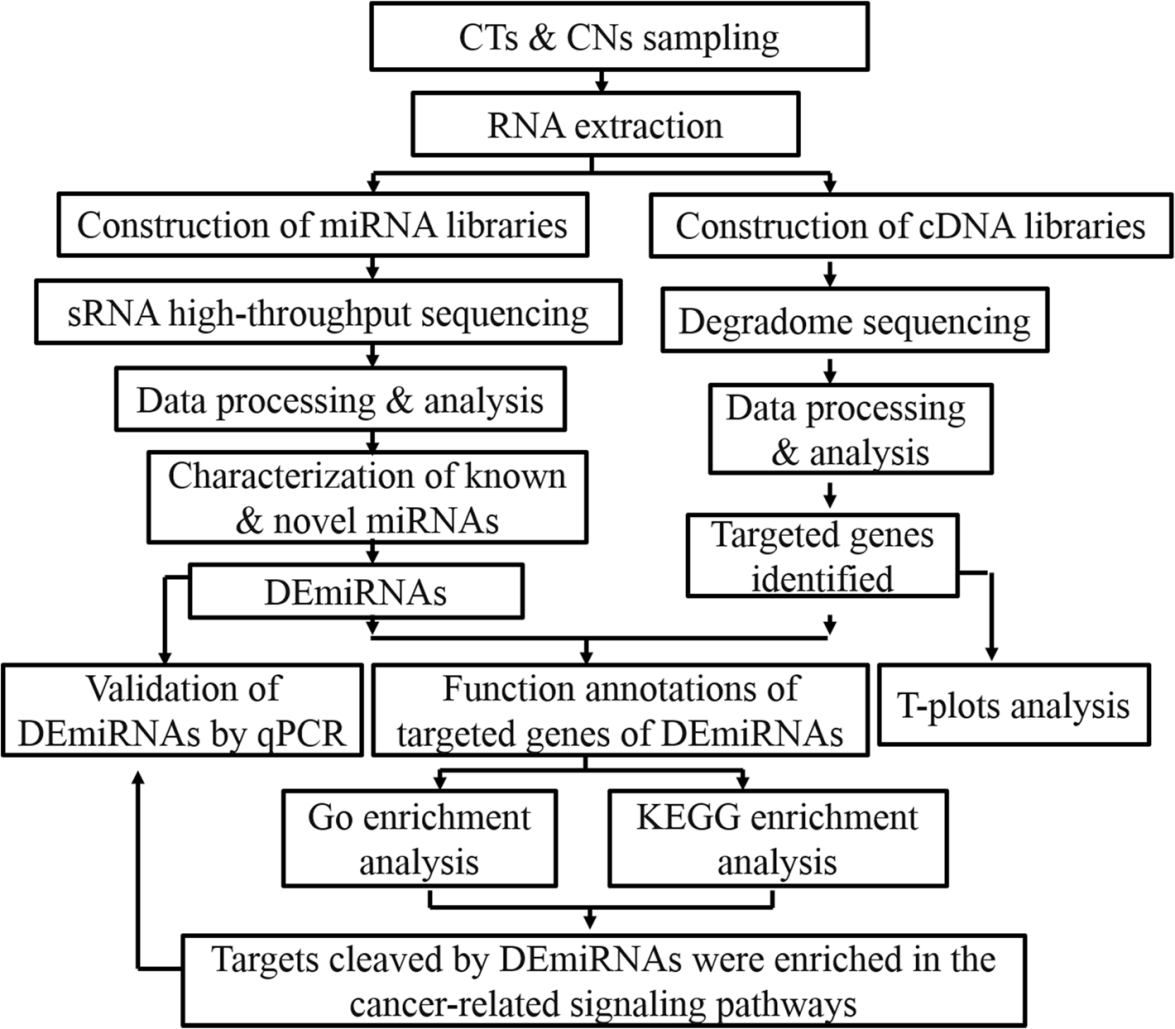
The flowchart of the study. High-throughput sRNA sequencing was employed to identify and profile miRNAs and DS was used to identify the cleaved target genes. After a series of data processing and bioinformatic analysis, targets cleaved by DEmiRNAs were found to be enriched in the cancer-related signaling pathways

Target genes corresponding to different expressed miRNAs (DEmiRNAs) were input into the Database for Annotation, Visualization and Integrated Discovery (DAVID) for gene ontology (GO) and Kyoto Encyclopedia of Genes and Genomes (KEGG) analysis. Fisher’s exact test was used for statistical analysis. GO terms and KEGG pathways with a corrected *p* ≤ 0.05 were regarded to be significantly enriched. The correlation between the expression of potential miRNA targets from cancer-related pathways and clinical features were investigated at cBioPortal. The flowchart of the study was presented in Fig. 1.

## Results

### High-throughput sequencing of sRNAs for CRC patients

Based on the sequencing data obtained from the CTs and CNs of six CRC patients, 12 sRNA libraries were constructed to identify miRNAs. Raw sRNA sequencing reads were deposited at NCBI (https://www.ncbi.nlm.nih.gov/) under accession number GSE125961. 79,012,674 and 83,084,696 unfiltered raw reads were obtained from the CT and CN libraries, respectively (Supplementary Table 3). The analysis yielded a total of 4,262,573 and 2,652,420 unique sequences, respectively. Subsequently, redundant data such as adaptor dimers and junk reads were excluded. Consequently, a total of 1,619,465 and 824,818 clean sRNA sequence reads remained. The percentages and numbers of different sRNAs are listed in Supplementary Table 3. It was noted that the length interval of unique reads between the CT and CN libraries both ranged from 18 to 26 nt, with 22 nt being the most predominant (Supplementary Figure 1).

### Identification of known and novel miRNAs

Known miRNAs in CRC were identified by comparing the raw sequencing data with human reference genome and miRBase. Subsequently, 1278 known miRNAs (clustered into 337 families) were characterized in all libraries (Supplementary Table 4). The expression levels of known miRNAs in each library were estimated via the normalization of their original reads as transcripts per million (TPM). An extremely variable frequency spectrums of miRNA were observed, ranging from 0 to > 1,000,000 TPM. The most abundant miRNAs in the CT and CN libraries were hsa-miR-143-3p_R + 1 and hsa-miR-143-3p_R + 1, respectively. In contrast, hsa-miR-381-5p_R + 1 and hsa-miR-383-5p showed low expression levels.

After removing known miRNAs, clean unique reads were employed for the identification of novel miRNAs in CRC. Ultimately, 131 novel miRNAs were detected in the 12 libraries (Supplementary Table 5). It was noted that the expression of most novel miRNAs identified exhibited relatively low, which was consistent with the results of previous researches in other human models [9].

### Differential expression analysis of known and novel miRNAs between CT and CN libraries

**Fig. 2:**
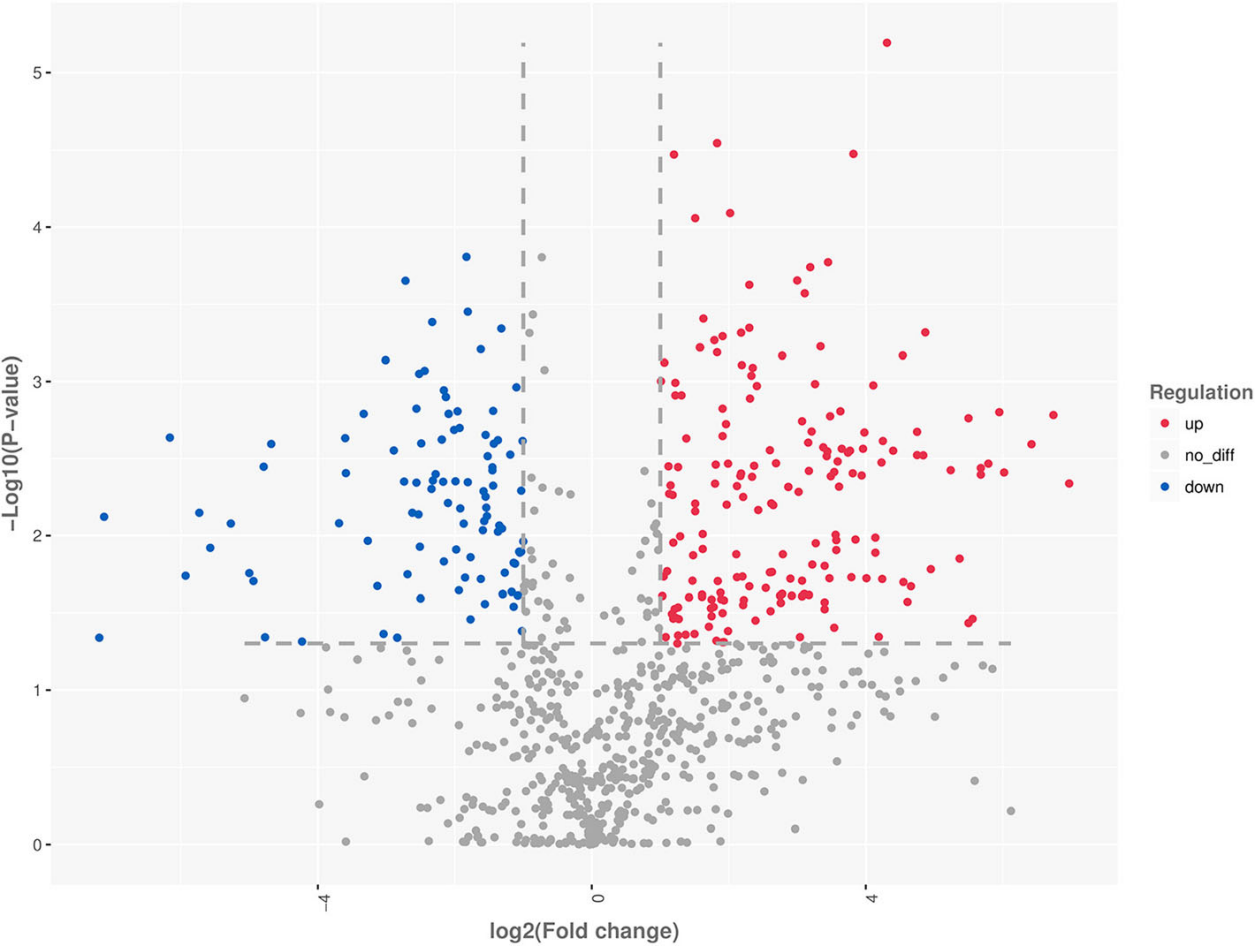
Volcanic diagrams showing the number of DEmiRNAs between CTs and CNs. The red dots indicate miRNAs with significant differences, and the blue dots indicate that miRNAs without significant differences

In total, 428 differentially expressed miRNAs (DEmiRNAs) between the CT and CN libraries were identified (293 and 135 were upregulated and downregulated, respectively) (Fig. 2). In detail, 420 of the 1090 known DEmiRNAs were detected in the libraries (Supplementary Table 6). Of these, 64, 171, and 185 were miRNAs yielded *p* value ≤0.001, ≤ 0.01, and ≤ 0.05, respectively. In detail, 51 miRNAs were up-regulated, whereas 13 were down-regulated (*p* ≤ 0.001); 109 miRNAs were up-regulated, whereas 62 were down-regulated (*p* ≤ 0.01); 125 miRNAs were up-regulated, whereas 60 were down-regulated (*p* < =0.05). Notably, hsa-miR-548au-5p_R-1 was the most significantly up-regulated miRNA with a *p*-value of 2.09E-10, whereas hsa-miR-1271-5p was the most significantly down-regulated miRNA with a *p*-value of 1.56E-04.

Eighty-one novel miRNAs were identified in the CT and CN libraries, the vast majority of which were expressed at a relatively low level (Supplementary Table 7). Only eight were differentially expressed and all of them were up-regulated, of which, PC-3p-30435_41 was the most significantly with a *p*-value as 7.77E-09 (Table 1).

**Table 1:** The top 10 significantly differentially expressed novel miRNAs between the CT and CN libraries

miR_name	miR_seq	Up/down	Fc	Log2(Fc)	***P*** value
PC-3p-30435_41	CTACCCCAGGATGCCAGCATAGTT	up	inf	inf	7.77E-09*
PC-3p-24565_58	ATGTTATGATGATGGGCGAAA	up	inf	inf	9.85E-08*
PC-5p-23775_61	TAGGGTGATGAAAAAGAAT	up	10.40	3.38	2.67E-03*
PC-5p-34972_32	CACCCTTTCCTGTGCCCTTTT	up	inf	inf	4.24E-03*
PC-5p-16080_103	TTAGTGGCTCCCTCTGCCTGCA	up	8.12	3.02	5.20E-03*
PC-5p-2366_1399	TTGCAAGCAACACTCTGTGGCAGA	up	6.19	2.63	1.72E-02*
PC-5p-37743_28	GTGGAGGACTGAGAAGGTGAGGC	up	3.68	1.88	2.34E-02*
PC-5p-31711_38	AATAATGATGATGACATACTGATA	up	inf	inf	2.52E-02*
PC-5p-22018_68	ATTCCCACTGTCCCTACCTAT	up	6.19	2.63	6.67E-02
PC-5p-77988_5	TCAGACACAGGTATGGCTGGCTCT	up	inf	inf	7.56E-02

*Inf* Infinity, *Fc* Fold change; *, *P* ≤ 0.05

DEmiRNAs were also characterized between the early-stage I-II (CT-E) and advanced-stage III-IV (CT-A) libraries to detect the CRC pathological grading-related miRNAs. However, only six DEmiRNAs were identified, of which, hsa-miR-10,527-5p, cgr-miR-1260_L + 1, hsa-miR-301b-5p, and dno-miR-450c-5p, hsa-miR-548az-5p_L-1R + 1 were upregulated, and only hsa-miR-874-3p_R + 1 was downregulated (Supplementary Table 8). None of any novel miRNAs belonged to DEmiRNAs.

### Validation of DEmiRNAs by qRT-PCR

**Fig. 3:**
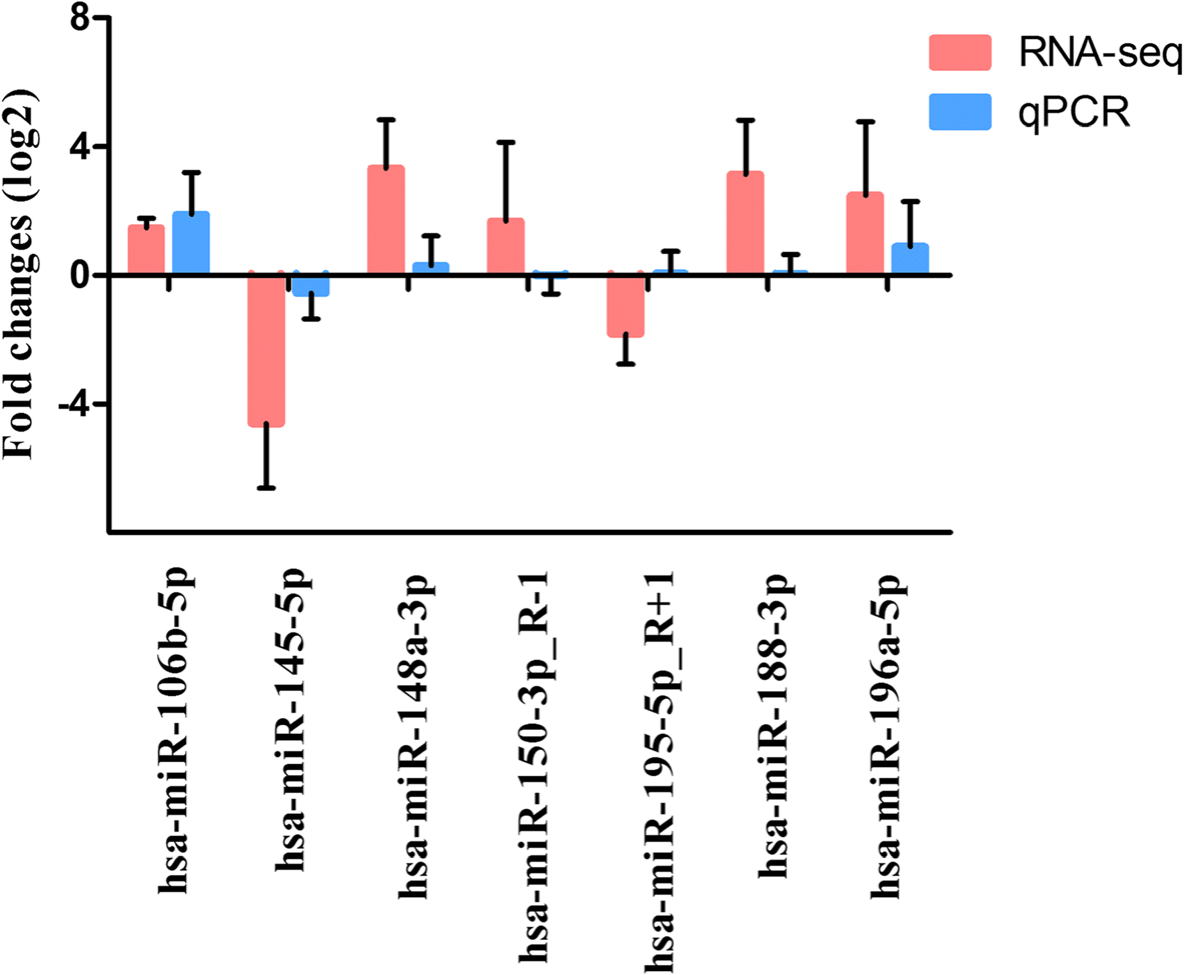
Comparisons of the expression levels of seven miRNAs between RNA-seq and qPCR. Fold change (log2) in CN libraries relative to CT libraries were detected by RNA-seq. The relative expression levels of miRNAs were normalized to the expression of U6 in qPCR

The expression of seven randomly selected miRNAs was validated using qRT-PCR to verify the high-throughput sequencing results. qPCR analysis indicated that, although the fold change of expression did not completely coincide with that measured using the sequencing data, the trend performed similarity (Fig. 3). For example, both the sequencing and qRT-PCR results confirmed the up-regulation of hsa-miR-106b-5p, hsa-mir-148a-3p and hsa-miR-196a-5p in the CT libraries compared with CN libraries, and the down-regulation of hsa-miR-145-5p in the CT libraries. These results indicated the reliability of high-throughput sequencing as a method to predict the expression of miNRAs.

### Identification of target genes of miRNAs using DS

Target genes of miRNAs in CRC were identified by DS at a global level and the raw sequencing data is uploaded at NCBI with accession number GSE125962. 29,604,631 raw reads were acquired from the libraries. Following the removal of the reads without the CAGCAG adaptor, 4,855,908 unique raw reads remained, which were subsequently mapped to the human genome database. 22,187,626 transcript-mapped reads were obtained and the mapped reads represented annotated human genes in the library.

**Fig. 4:**
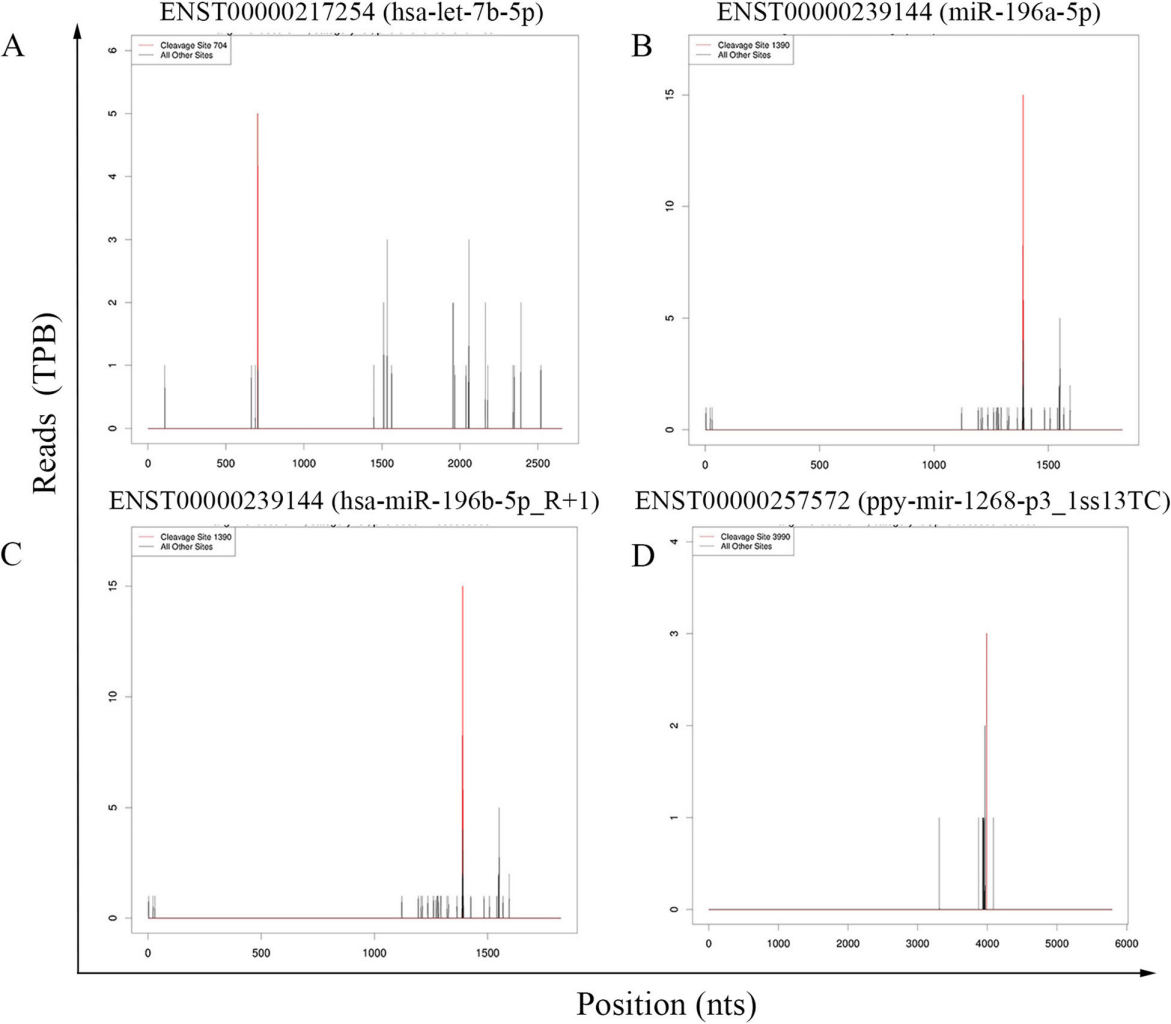
Examples of T-plots of miRNA targets identified by DS. The red lines indicate miRNA-mediated cleavage. **a** hsa-let-7b-5p slicing ENST00000217254 at 704; **b** miR-196a-5p slicing ENST00000239144 at 1390; **c** hsa-miR-196b-5p_R + 1 slicing ENST00000239144 at 1390; **d** ppy-mir-1268-p3_1ss13TC slicing ENST000002575724 at 3990

A potential cleaved target was defined based on the algorithm of the CleaveLand pipeline. In total, 9685 transcripts containing 2797 target genes were characterized as targets for 268 known miRNAs. Of those, 110, 112, 2261, 423, and 6779 were categorized into subgroups 0, 1, 2, 3, or 4 in the libraries (Supplementary Table 9). The T-plots for some targets are illustrated in Fig. 4. Among the 268 known miRNAs, some target only one transcript (eg, bta-miR-12034_1ss19TG), whereas the others have multiple targets (bta-miR-11987_L-1_1ss8TA). In addition, we noticed that the majority of targets were anchored in protein coding locus, which had been previously reported in certain plants [3]. Of the 9685 transcripts, only 358 (3.70%) target sites located in untranslated region (Table 2). Functional analysis indicated that these target genes were concentrated in mRNA transport, endocytosis, early endosome to late endosome transport, protein phosphorylation, etc.

**Table 2:** The locations of transcripts targeted by known and novel miRNAs

miRNAs	Degradome categories	Protein coding regions (N / %)	Untranslated regions (N / %)	Total (N)
**Known**	0	106 (96.36%)	4 (3.64%)	110
	1	103 (91.96%)	9 (8.04%)	112
	2	2159 (95.49%)	102 (4.51%)	2261
	3	413 (97.64%)	10 (2.36%)	423
	4	6546 (96.56%)	233 (3.44%)	6779
	total	9327 (96.30%)	358 (3.70%)	9685
**Novel**	0	1 (100.00%)	0 (0.00%)	1
	1	0 (NA)	0 (NA)	0
	2	63 (98.44%)	1 (1.56%)	64
	3	1 (33.33%)	2 (66.67%)	3
	4	128 (96.97%)	4 (3.03%)	132
	total	193 (96.50)	7 (3.50%)	200

*NA* not applicable

**Fig. 5:**
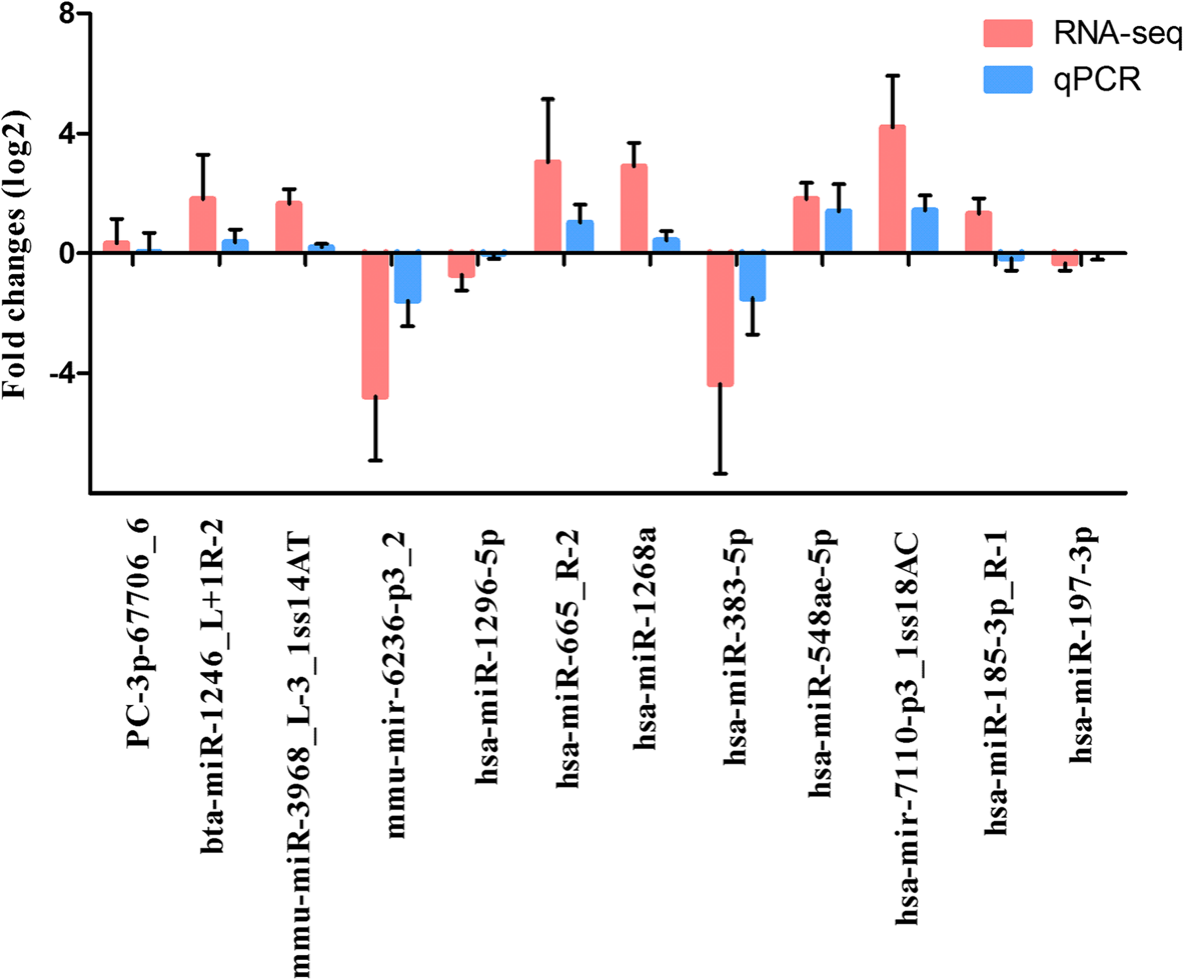
Comparisons of the expression levels of 12 miRNAs between RNA-seq and qPCR. Fold change (log2) in CN libraries relative to CT libraries were detected by RNA-seq. The relative expression levels of miRNAs were normalized to the expression of U6 in qPCR

For novel miRNAs, 200 transcripts covering 47 target genes were identified as targets for 33 novel miRNAs. Of those, 1, 0, 64, 3, and 132 transcripts were categorized into subgroups 0, 1, 2, 3, or 4 in the libraries (Table 2, Supplementary Table 10). Most of these novel miRNAs belonged to categories 2 and 4. Similarly, only 7 (3.50%) target sites located in untranslated region. And among the 33 novel miRNAs, some have only one targeted transcript, whereas the others were polygamous. Among the latter group of miRNAs, PC-3p-67706_6 exhibited the highest number of targets (20 transcripts). Both the high-throughput sequencing and qPCR results confirmed that the levels of PC-3p-67706_6 was up-regulated in the CT libraries compared with CN libraries (Fig. 5). Functional descriptions of these target genes were also conducted; however, fewer functions were detected using the GO analysis.

For six DEmiRNAs between CT-E and CT-A, we found that only the miRNA cgr-miR-1260_L + 1 had nine transcripts covering three target genes.

### Function analysis of the potential miRNA targets

Among the known miRNAs, 263 target genes for 84 of the 420 DEmiRNAs were validated using DS. However, for novel miRNAs, only one DEmiRNA (PC-5p-23775_61), was identified to direct cleaver the target gene. The whole of 264 target genes for these 84 DEmiRNAs were subjected to Fisher’s exact test-based GO biological and functional analysis to investigate the processes involved in CRC development and progression (Supplementary Table 11). The results of the functional analysis of miRNAs were generated using DAVID. According to number of genes involved, we listed the top eight significantly related GO terms in Table 3. The results indicated that in the biological process (BP), the targeted genes were mainly enriched in membrane protein proteolysis, regulation of nucleic acid-templated transcription, mRNA processing, negative regulation of non-canonical Wnt signaling pathway, negative regulation of transforming growth factor beta receptor (TGF-β) signaling pathway, negative regulation of TOR signaling, etc. In the cellular component (CC) analysis, the genes were concentrated in nucleoplasm, lysosomal membrane, membrane, nuclear membrane and spindle pole centrosome. For molecular function (MF), the up-regulated genes were mainly anchored in protein binding, poly(A) RNA binding, ATPase activity, actin binding, enzyme binding, etc. The results implied biological functions of miRNAs in the regulation of genes of CRC.

**Table 3:** Go analysis of mRNAs targets of DEmiRNAs

Term	Count	***P*** Value^**a**^
**GO-BPs**		
GO:0033619~membrane protein proteolysis	3	0.005678
GO:1903506~regulation of nucleic acid-templated transcription	3	0.014466
GO:0006397~mRNA processing	7	0.018271
GO:2000051~negative regulation of non-canonical Wnt signaling pathway	2	0.034437
GO:0030512~negative regulation of transforming growth factor beta receptor signaling pathway	4	0.038329
GO:0032007~negative regulation of TOR signaling	3	0.04162
GO:0008380~RNA splicing	6	0.044499
GO:0000398~mRNA splicing, via spliceosome	7	0.045339
**GO-CCs**		
GO:0005654~nucleoplasm	57	8.50E-06
GO:0005765~lysosomal membrane	11	0.001198
GO:0016020~membrane	41	0.001693
GO:0031965~nuclear membrane	9	0.004743
GO:0031616~spindle pole centrosome	3	0.005441
GO:0000118~histone deacetylase complex	4	0.007827
GO:0005925~focal adhesion	11	0.014141
GO:0030660~Golgi-associated vesicle membrane	3	0.023398
**GO-MFs**		
GO:0005515~protein binding	134	4.51E-05
GO:0044822~poly(A) RNA binding	30	7.93E-05
GO:0016887~ATPase activity	9	0.00165
GO:0003779~actin binding	11	0.001948
GO:0019899~enzyme binding	10	0.018955
GO:0005102~receptor binding	10	0.026515
GO:0003743~translation initiation factor activity	4	0.036608
GO:0004583~dolichyl-phosphate-glucose-glycolipid alpha-glucosyltransferase activity	2	0.047016

^a^*BP* biological process, *MF* molecular function, *CC* cellular component. *P* value < 0.05 was considered as threshold values of significant difference

A KEGG pathway analysis was conducted to recognize the signaling pathways involved in the progression of CRC. The eight KEGG pathways identified in our study are listed in Table 4. Among these, the proteoglycans in cancer and AMPK signaling pathways were cancer-related. Actin gamma 1 (ACTG1), DEAD-box helicase 5 (DDX5), ezrin (EZR), fibronectin 1 (FN1), hematopoietic cell-specific Lyn substrate 1 (HCLS1), phosphoinositide-3-kinase regulatory subunit 5 (PIK3R5) and SOS Ras/Rho guanine nucleotide exchange factor 2 (SOS2) were the potential miRNA targets in proteoglycans in cancer, AKT1 substrate 1 (AKT1S1), cystic fibrosis transmembrane conductance regulator (CFTR), PIK3R5, protein kinase AMP-activated non-catalytic subunit beta 1 (PRKAB1) and protein phosphatase 2 regulatory subunit Bgamma (PPP2R2C) were the potential miRNA targets in the AMPK signaling pathway, and actin gamma 1 (ACTG1) and symplekin (SYMPK) were part of the potential miRNA targets in the tight junction pathway. Also, we verified the expression of these 11 miRNA targeted genes using qRT-PCR (Fig. 5). Both the high-throughput sequencing and qRT-PCR results confirmed that the levels of bta-miR-1246_L + 1R-2, mmu-miR-3968_L-3_1ss14AT, hsa-miR-665_R-2, hsa-miR-1268a, hsa-miR-548ae-5p and hsa-mir-7110-p3_1ss18AC were up-regulated in the CT libraries compared with CN libraries, whereas that of mmu-mir-6236-p3_2, hsa-miR-383-5p and hsa-miR-197-3p was down-regulated in the CT libraries. The above results further validated the reliability of the high-throughput sequencing as a method to predict the expression of miNRAs.

**Table 4:** KEGG pathway analysis of mRNAs targets of DEmiRNAs

KEGG pathways	Count	***P*** Value^*^
hsa04530:Tight junction	6	0.006847
hsa05146:Amoebiasis	6	0.015314
hsa04510:Focal adhesion	8	0.023369
hsa04810:Regulation of actin cytoskeleton	8	0.025633
hsa03015:mRNA surveillance pathway	5	0.036679
hsa05205:Proteoglycans in cancer	7	0.047755
hsa04670:Leukocyte transendothelial migration	5	0.074216
hsa04152:AMPK signaling pathway	5	0.089858

*P* value < 0.05 was considered as threshold values of significant difference

**Fig. 6:**
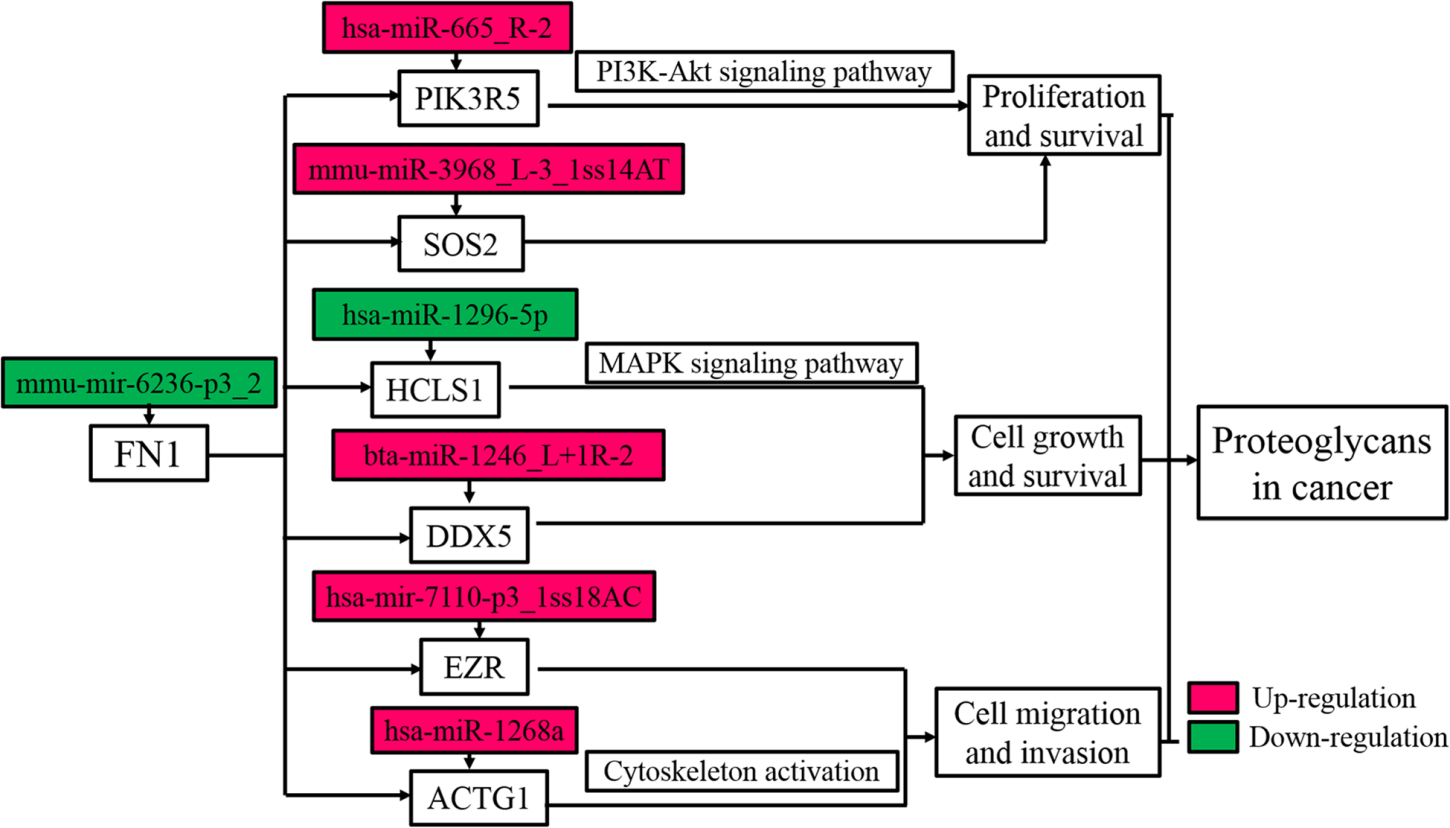
KEGG pathway analysis of targeted genes involved into proteoglycans in cancer pathway. mmu-mir-6236-p3_2 targeted the FN1 gene; hsa-miR-665_R-2, was involved in proliferation and survival, and controlled the accumulation level of the PIK3R5 protein in the PI3K-Akt signaling pathway; hsa-miR-1296-5p targeted the HCLS1 gene, to respond to the MAPK signaling pathway. Red rectangle represents up-regulated targets and blue rectangle represents down-regulated targets

Non-cancer related pathways, such as tight junction, amoebiasis, focal adhesion, regulation of actin cytoskeleton, and mRNA surveillance pathway were also enriched. Examples of targeted genes involved into proteoglycans in cancer pathway and the corresponding miRNAs were exhibited in Fig. 6. These results exerted that miRNAs played a vital role in the regulation of CRC initiation, progression, and metastasis by affecting the proteoglycans in cancer pathway.

### Clinical significance of miRNA targets in proteoglycans in cancer and the AMPK signaling pathways

**Fig. 7:**
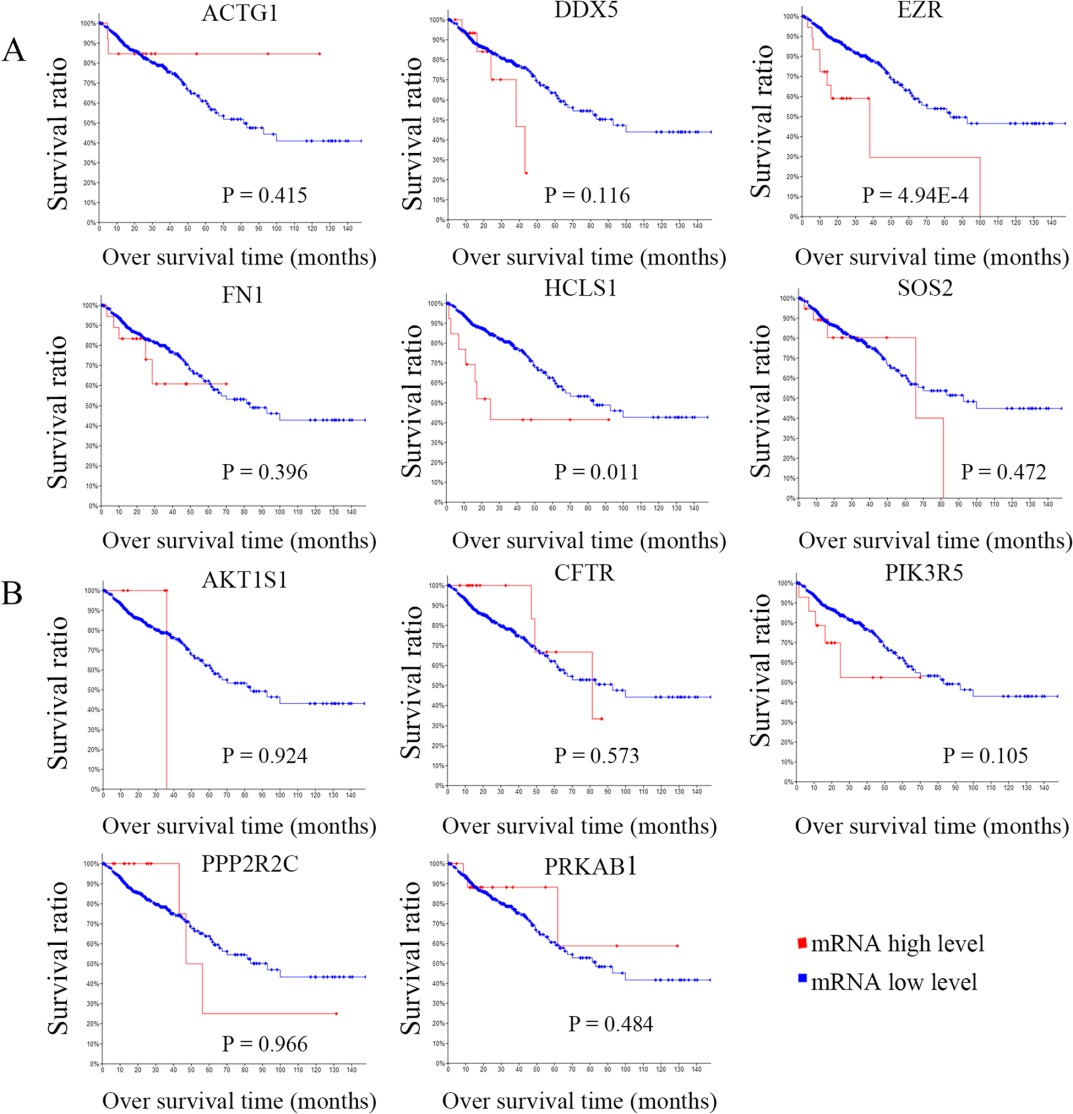
Analysis of the correlation between CRC prognosis (OS) and the expression of miRNA targeted genes in cancer-related pathways. **a** targets regulating proteoglycans; **b** targets regulating the AMP-activated protein kinase signaling pathway

**Fig. 8:**
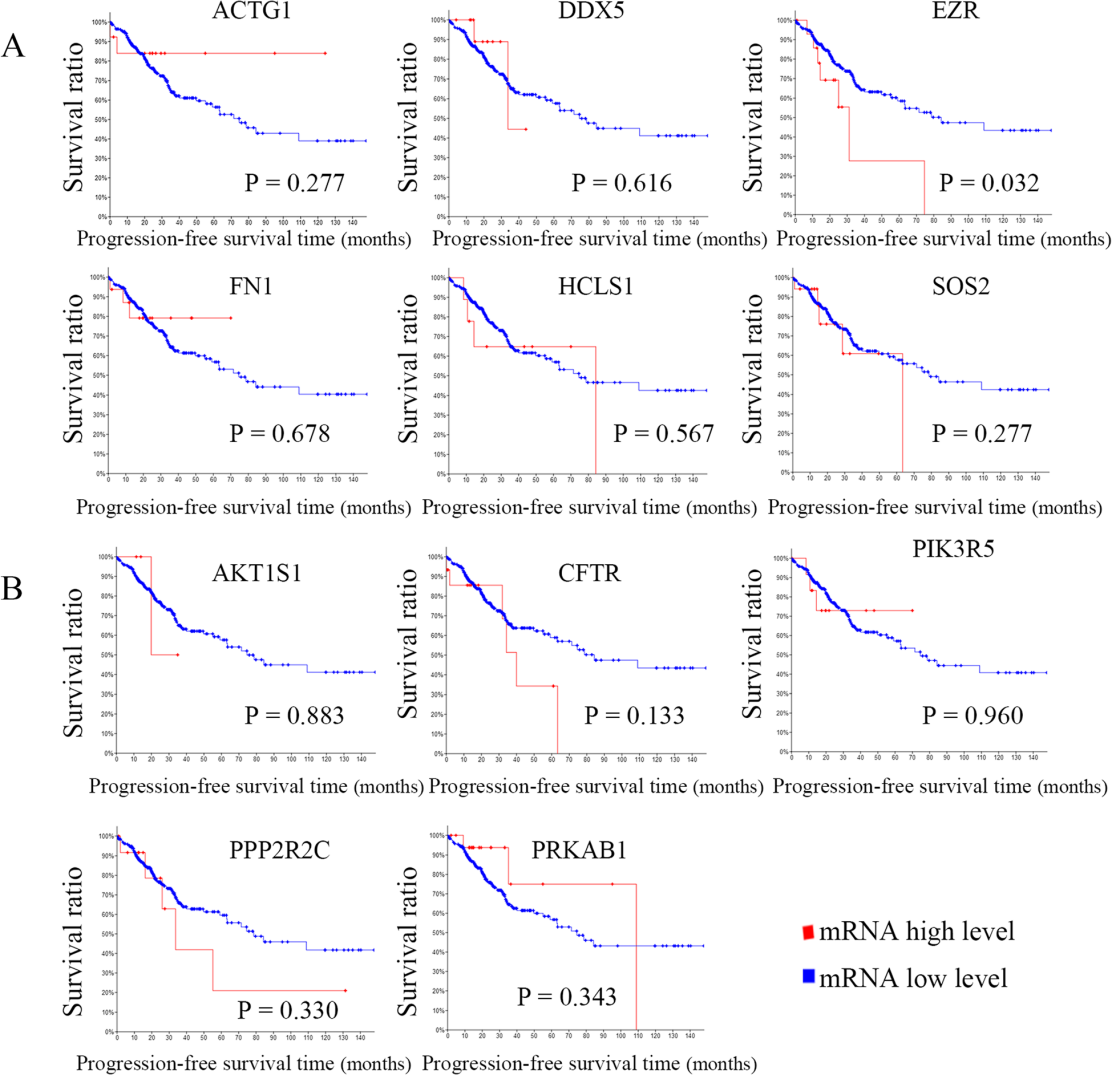
Analysis of the correlation between CRC prognosis (PFS) and the expression of miRNA targeted genes in cancer-related pathways. **a** targets regulating proteoglycans; **b** targets regulating the AMP-activated protein kinase signaling pathway

To identify potential targeted genes with prognostic characteristics, the CRC dataset of TCGA with larger 382 tissue samples were interrogated to profile the mRNA expression levels of targeted genes using survival Kaplan-Meier estimate at cBioPortal. As a result, using Onco Query Language (OQL) EXP > 2, in proteoglycans in cancer signaling pathway, two targets EZR and HCLS1 were found to be negatively correlated with overall survival (OS) (*P* = 4.94E-4 and 0.011) (Fig. 7a). However, none of targets were found be significantly associated with OS in the AMP-activated protein kinase signaling pathway (Fig. 7b). Recurrence is a major concern for cancer survivors. The results indicated that EZR was negatively correlated with progression-free survival (PFS) in proteoglycans in cancer signaling pathway (*P* = 0.032) (Fig. 8a). On the contrary, none of targets were found be significantly associated with PFS in the AMP-activated protein kinase signaling pathway (Fig. 8b).

**Fig. 9:**
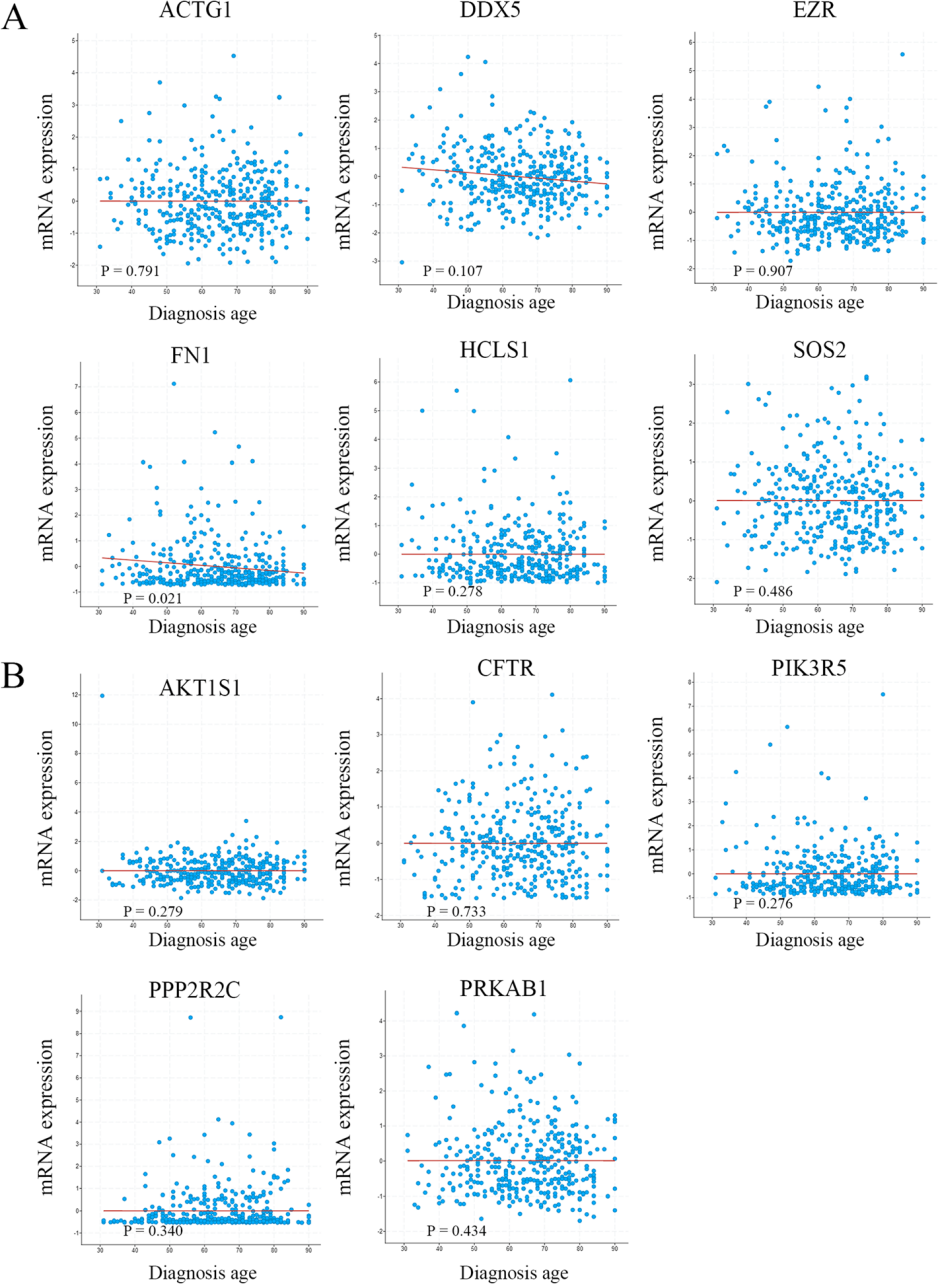
Analysis of the correlation between CRC diagnosis age and the expression of miRNA targeted genes in cancer-related pathways. **a** targets regulating proteoglycans; **b** targets regulating the AMP-activated protein kinase signaling pathway

To reveal the clinical significance of these 11 miRNA targets, mRNA expression alterations, as well as alterations to other clinical features, such as sex, age in diagnosis, tumor stage, histologic type, metastatic status and KRAS mutation, were investigated. However, apart from FN1 being identified to be associated with diagnosis age (*P* = 0.021) (Fig. 9), other clinical features, including sex, tumor stage, histologic type, metastatic status and KRAS mutation, were evaluated separately, and no significant associations with 11 miRNA targets were observed (Supplementary Figs. 2, 3 and 4).

## Discussion

sRNAs, including miRNAs, affect the post-transcription of target genes via multiple mechanisms, including deadenylation or decapping. These functions result in mRNA destabilization by disturbing ribosome recruitment, as well as degradation by direct cleavage of target RNAs [6, 7]. Considering that only Ago2 is characterized in controllable catalytic activity, miRNA-induced mRNA cleavage happens occasionally in animals [11]. The development of DS renders the observation and identification of miRNA-mediated cleavage events more feasible. Jain et al. used DS to reveal 29 cleaved targets of insect miRNAs from malaria host *Anopheles stephensi* [8]. Ashley et al. found 398 sciRNAs and 810 cleaved target genes in various organs of mouse [13].

In CRC, The involvement of miRNAs in the regulation of gene post-transcription via mRNA translational inhibition or destabilization are well documented [19, 20]. However, how they function to cleave target mRNAs remain to be elucidated. Herein, we used high-through sequencing and DS technologies to investigate the profile of miRNA-induced mRNA cleavage in CRC. A total of 1278 known and 131 novel miRNAs were identified from the CT and CN libraries, respectively. Certain established miRNAs involved in vital signaling pathways, such as miR-1271 and miR-490-3p, were identified. Wnt signals play important roles in the regulation of cancer cell proliferation and migration. Sun et al. reported that miRNA-1271 negatively regulated metadherin & Wnt signaling to inhibit cell proliferation and invasion of CRC [23]. Zheng et al. indicated that miR-490-3p worked as a suppressor to mediate CRC progression via the control of the Wnt/β-catenin signaling pathway [24]. Moreover, miR-17, miR-21 and miR-106a have been identified to regulate TGF-β receptor 2, an important member of the TGF-β family. In addition, some miRNAs, such as PC-5p-16080_103 and PC-3p-31923_38, were newly identified. Notably, the majority of these novel miRNAs (except PC-3p-3445_793 and PC-5p-2366_1399) had relatively low expression (less than 100 TPM), This observation has also reported in certain plants [3, 25]. The above results implied that the expression levels of highly conserved miRNAs was higher than these of less conserved miRNAs. This may partly be attributed to the critical roles of these conserved miRNAs in human evolution. Although most novel miRNAs showed at a low expression levels, their potential roles in CRC carcinogenesis, development, and metastasis remained to be deserved to be investigated.

Four hundred twenty known DEmiRNAs were detected between CT and CN libraries. Of those, 285 DEmiRNAs were up-regulated. Among the DEmiRNAs, miR-21 is an established miRNA, which is upregulated in CRC and is speculated to be associated with tumor stage and outcome [26]. Notably, 135 known DEmiRNAs were downregulated. Among those, miR-143 was proposed to downgrade in colon cancer and negatively correlated with tumor size and disease-free survival. Moreover, it has the potential for KRAS wild-type CRC prognosis [27, 28]. The target gene of miR-143 encodes the cysteine and serine rich nuclear protein 3 (CSRNP3), single nucleotide polymorphisms (SNPs) of which has been identified as a novel susceptibility mark for metabolic syndrome [29].

DS provides a powerful tool for the investigation of miRNA-mRNAs interactions at a global level. Herein, a total of 9685 potential target transcripts including 2797 genes were characterized as targets for 268 known miRNAs. The functions of the identified targets of known miRNAs were diverse. For example, hsa-mir-5684-p3_1ss18CG targeting major vault protein (MVP), which was found to be regulated by Notch1 and contribute to overcome chemoresistance in triple-negative breast cancer cells [30]. hsa-miR-5585-3p_L-3R-1_1ss5AG and hsa-mir-7851-p5_1ss12AC targeting AKT serine/threonine kinase 3 (AKT3) played swinging roles in the tumorigenesis, development, and progression of various types of cancer, including CRC [31–33]. DS identified a total of 202 potential target transcripts, covering 47 target genes for 33 novel miRNAs. Compared with known miRNAs, targets of novel miRNAs were discrete and GO analysis did not identify related functions. These findings implied the young and unstable evolution of these novel miRNAs.

It was noted that DemiRNAs were also identified between the early-stage I-II (CT-E) and advanced-stage III-IV (CT-A) libraries. However, only six DEmiRNAs were detected and the only miRNA cgr-miR-1260_L + 1 had nine transcripts covering three target genes. The low number of DEmiRNAs identified may be due to the limitation of sample number used and further study are considered to add more cases to objectively draw the profile of miRNA.

A total of 264 target genes of 85 DEmiRNAs were validated using DS. Among those, only one novel DEmiRNAs (PC-5p-23775_61), was identified to direct cleave the target the gene lysine demethylase 1 (KDM1B). KDM1B regulated histone lysine methylation and acted as an epigenetic marker regulating gene expression and chromatin function [34]. In addition, KDM1B was also reported to be targeted by miR-215 to mediate glioma-initiating cells to adapt to hypoxia [35]. Functional analysis indicated that these potential miRNA targets were enriched in the proteoglycans in cancer and AMPK signaling pathway. Seven target genes, namely DDX5, SOS2, ACTG1, EZR, FN1, HCLS1 and PIK3R5, were involved in proteoglycans in cancer signaling pathway. mmu-mir-6236-p3_2 targeted the FN1 gene, and hsa-miR-1296-5p targeted the HCLS1 gene, to respond to the MAPK signaling pathway. Another miRNA, hsa-miR-665_R-2, was involved in proliferation and survival, and regulated the expression of the PIK3R5 protein in the PI3K-Akt signaling pathway. The expression of these miRNA targets was validated by qPCR, which was consistent with high-throughput sequencing data (Fig. 5). Moreover, five target genes, namely hsa-miR-1268a-mediated cleavage of AKT1S1, hsa-miR-383-5p-mediated cleavage of CFTR, hsa-miR-665_R-2-mediated cleavage of PIK3R5, hsa-miR-548ae-5p-mediated cleavage of PRKAB1, and hsa-mir-7110-p3_1ss18AC-mediated cleavage of PPP2R2C, were involved in the AMPK signaling pathway. These findings indicated that the vast majority of miRNAs functioned at different manners to regulate CRC by guiding the cleavage of target genes, which attracted us to explore the clinical signification of these miRNA targets.

Since the number of patient samples was limited, we interrogated the CRC dataset of TCGA at cBioPortal to investigate the relationship between miRNA targets in cancer-related signal pathways and clinical features. After systemic analysis of prognosis-related miRNA targets in cancer-related signal pathways, interestingly, we found that EZR and HCLS1 had the potential prognostic characteristics with CRC regarding OS or recurrence, which drove us to further investigate the correlation between them and other clinical features. However, apart from FN1 being identified to be associated with diagnosis age, other clinical features, including sex, tumor stage, histologic type, metastatic status and KRAS mutation, were evaluated separately, and no significant associations with 11 miRNA targets were observed.

## Conclusion

In this study, a systematic and comprehensive evaluation of CRC-related miRNAs and their endonucleolytic cleaved target genes was conducted through high-throughput sequencing and DS. The present findings support previous evidence showing that mRNA cleavage guided by miRNAs occurs in animal. Furthermore, some miRNAs may have the potential as prognosis markers of CRC. These findings promoted and improved the better appreciation of the novel interaction mode between miRNAs and target genes in CRC .

## Supplementary information

**Additional file 1: Table S1.** Clinical characteristics of the study population. **Table S2.** The primers of miRNAs validated by qRT-PCR. **Table S3.** Annotation of sRNAs sequences. **Table S4.** All Expressed known miRNA. **Table S5.** All novel or predicted candidate miRNAs (PC miRNAs) in this study. **Table S6.** Differentially expressed known miRNAs between the CT and CN libraries. **Table S7.** Differentially expressed novel miRNAs between the CT and CN libraries. **Table S8.** Differentially expressed miRNAs between the early-stage I-II (CT-E) and advanced-stage III-IV (CT-A) libraries. **Table S9.** All targets of known miRNAs. **Table S10.** All targets of novel miRNAs. **Table S11.** Potential target genes of DEmiRNAs.

**Additional file 2: Figure S1.** The distribution of the lengths of miRNAs in CT and CN libraries.

**Additional file 3: Figure S2.** Analysis of the correlation between CRC tumor stage and the expression of miRNA targeted genes in cancer-related pathways. (A) targets regulating proteoglycans; (B) targets regulating the AMP-activated protein kinase signaling pathway.

**Additional file 4: Figure S3.** Analysis of the correlation between CRC histologic type and the expression of miRNA targeted genes in cancer-related pathways. (A) targets regulating proteoglycans; (B) targets regulating the AMP-activated protein kinase signaling pathway.

**Additional file 5: Figure S4.** Analysis of the correlation between CRC KRAS mutation and the expression of miRNA targeted genes in cancer-related pathways. (A) targets regulating proteoglycans; (B) targets regulating the AMP-activated protein kinase signaling pathway.

## Data Availability

The datasets generated and/or analyzed during the current study are available in the GEO repository, accession no. GSE125961 and GSE125962.

## Abbreviations

CRC: colorectal cancer
miRNAs: microRNAs
sRNA: small RNA
DS: degradome sequencing
GO: gene ontology
KEGG: Kyoto Encyclopedia of Genes and Genomes
DEmiRNAs: differentially expressed miRNAs
RT-PCR: reverse transcription-polymerase chain reaction
DAVID: Database for Annotation, Visualization and Integrated Discovery
TPM: transcripts per million
NCBI: National Center for Biotechnology Information
BP: biological process
MF: molecular function
CC: cellular component
mESCs: mouse embryonic stem cells
MVP: major vault protein
AKT3: AKT serine/threonine kinase 3
KDM1B: lysine demethylase 1
DDX5: DEAD-box helicase 5
SOS2: SOS Ras/Rho guanine nucleotide exchange factor 2
ACTG1: actin gamma 1
EZR: ezrin
FN1: fibronectin 1
HCLS1: hematopoietic cell-specific Lyn substrate 1
PIK3R5: phosphoinositide-3-kinase regulatory subunit 5
AKT1S1: AKT1 substrate 1
CFTR: cystic fibrosis transmembrane conductance regulator
PRKAB1: protein kinase AMP-activated non-catalytic subunit beta 1
PPP2R2C: rotein phosphatase 2 regulatory subunit B gamma
SNPs: single nucleotide polymorphisms
