# Supplementary figures and images for: Identification of microRNAs and their Endonucleolytic Cleavaged target mRNAs in colorectal cancer
Source: BMC Cancer. 2020 Mar 23;20:242. doi: 10.1186/s12885-020-06717-4 (PMC7092451; doi:10.1186/s12885-020-06717-4)

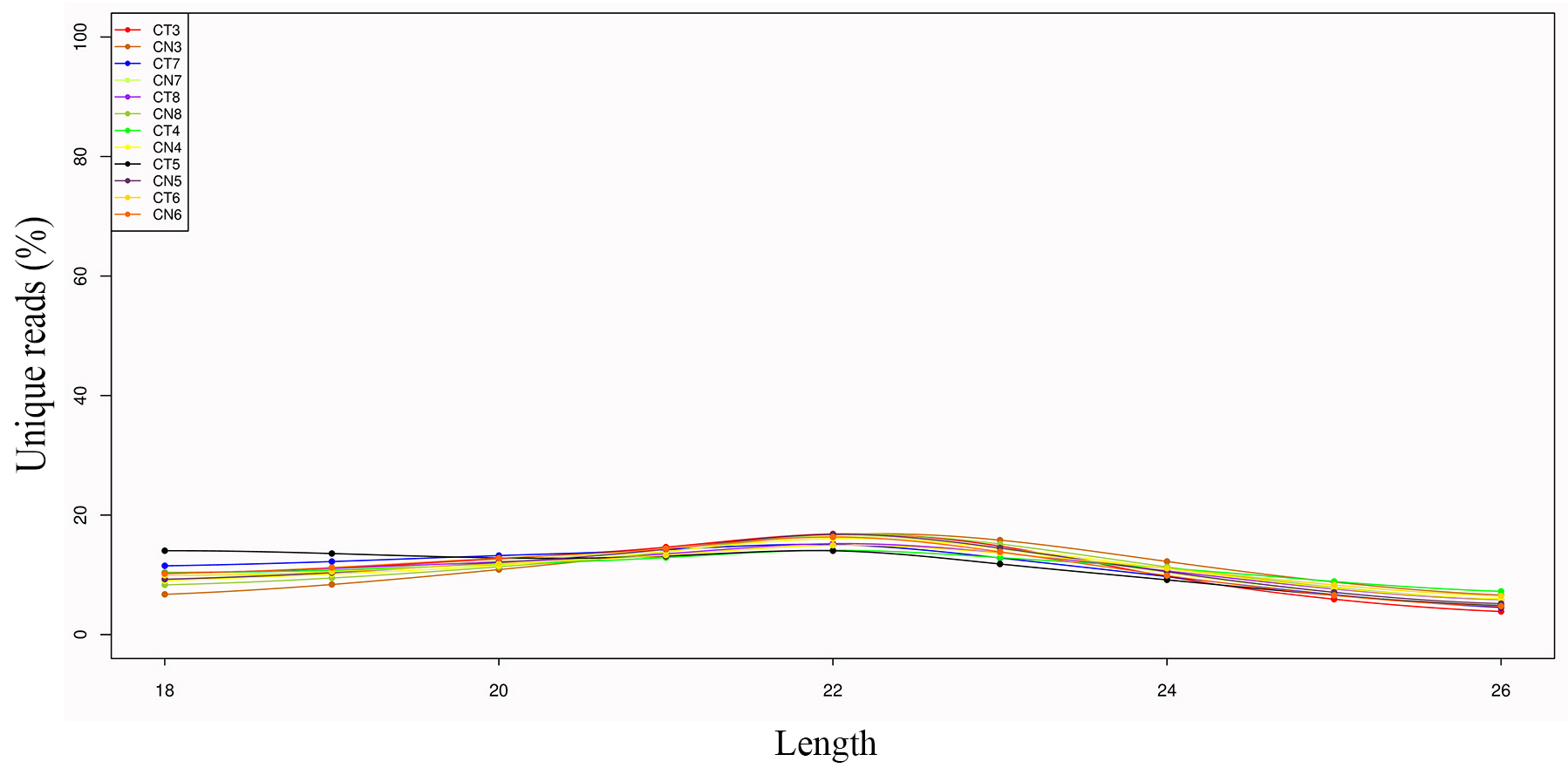

Supplement: Supplementary file 2 — Additional file 2: Figure S1. The distribution of the lengths of miRNAs in CT and CN libraries. [file 12885_2020_6717_MOESM2_ESM.tif]

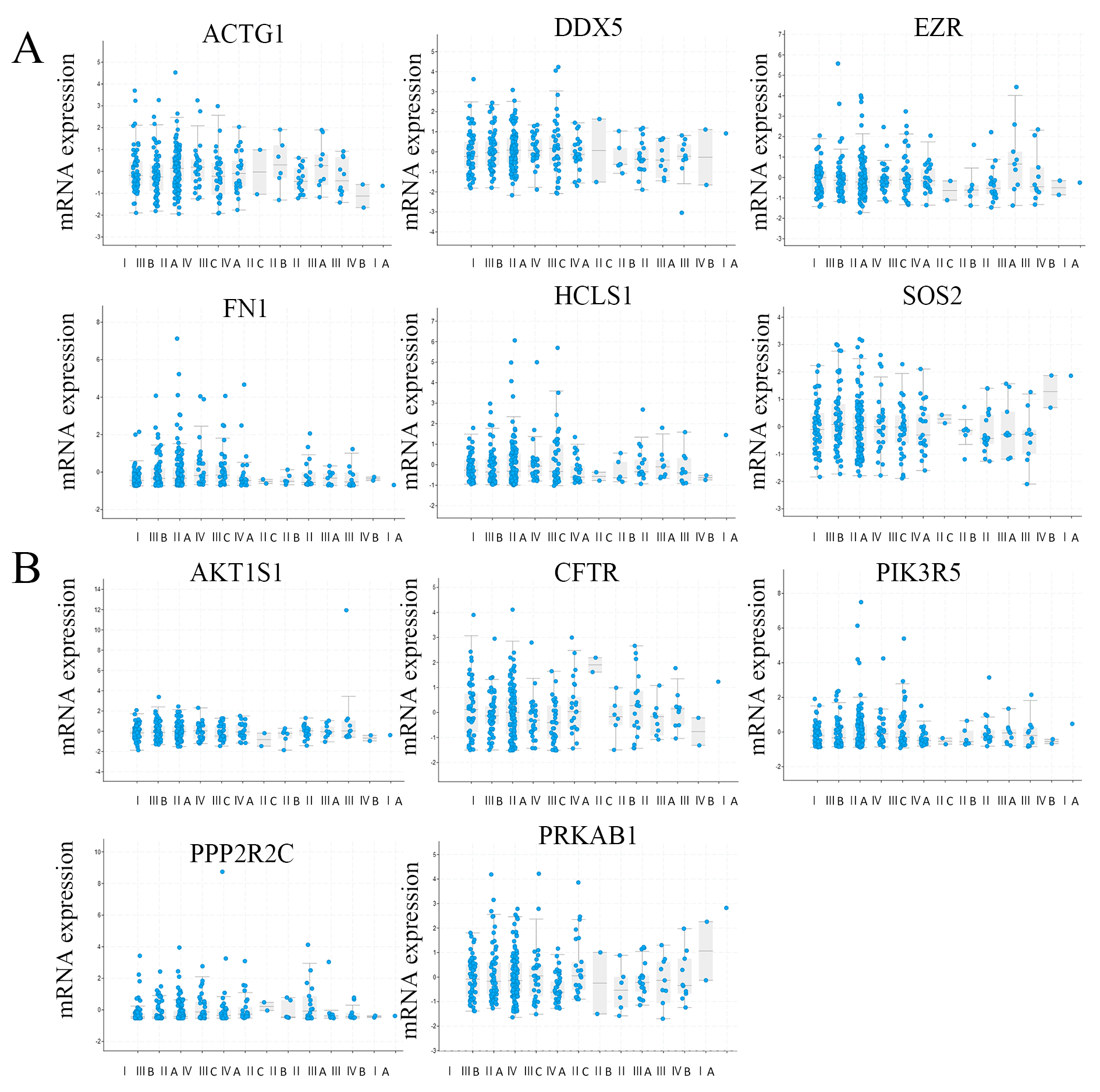

Supplement: Supplementary file 3 — Additional file 3: Figure S2. Analysis of the correlation between CRC tumor stage and the expression of miRNA targeted genes in cancer-related pathways. (A) targets regulating proteoglycans; (B) targets regulating the AMP-activated protein kinase signaling pathway. [file 12885_2020_6717_MOESM3_ESM.tif]

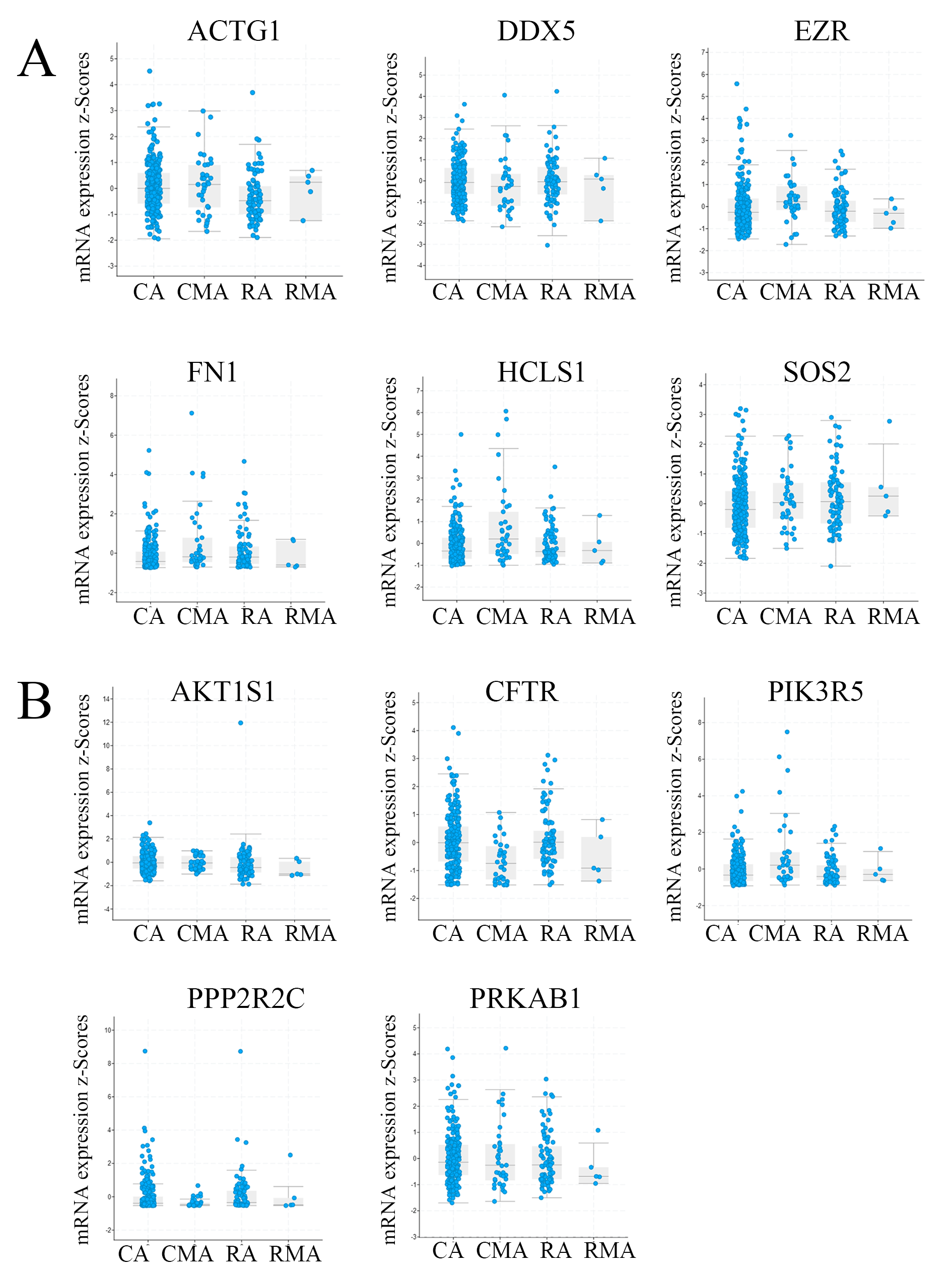

Supplement: Supplementary file 4 — Additional file 4: Figure S3. Analysis of the correlation between CRC histologic type and the expression of miRNA targeted genes in cancer-related pathways. (A) targets regulating proteoglycans; (B) targets regulating the AMP-activated protein kinase signaling pathway. [file 12885_2020_6717_MOESM4_ESM.tif]

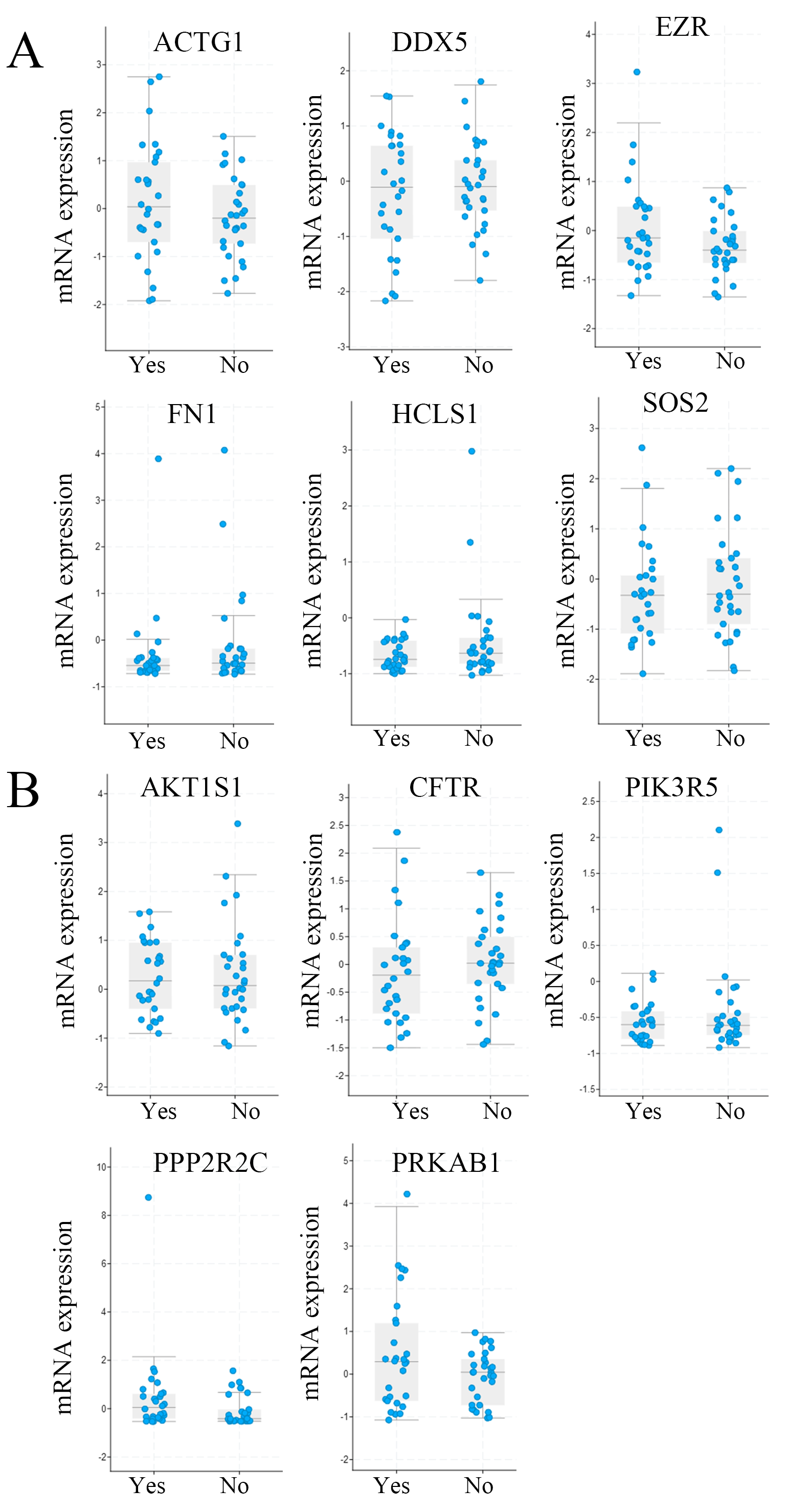

Supplement: Supplementary file 5 — Additional file 5: Figure S4. Analysis of the correlation between CRC KRAS mutation and the expression of miRNA targeted genes in cancer-related pathways. (A) targets regulating proteoglycans; (B) targets regulating the AMP-activated protein kinase signaling pathway. [file 12885_2020_6717_MOESM5_ESM.tif]
